# Effects of Endocrine Disrupting Chemicals on Fetal Weight: Exposure Monitoring Among Mothers with Gestational Diabetes Mellitus and Their Fetuses

**DOI:** 10.3390/ijms26094226

**Published:** 2025-04-29

**Authors:** Subeen Hong, Sae Kyung Choi, Jeong Ha Wie, Jae Eun Shin, Yun Sung Jo, Yeon Hee Kim, Byung Soo Kang, Oyoung Kim, Sangeun Won, Hee Ju Yoon, Hyeon Soo Kim, In Yang Park, Mihi Yang, Hyun Sun Ko

**Affiliations:** 1 Department of Obstetrics and Gynecology, Seoul St. Mary’s Hospital, College of Medicine, The Catholic University of Korea, Seoul 06591, Republic of Korea; 2 Department of Obstetrics and Gynecology, Incheon St. Mary’s Hospital, College of Medicine, The Catholic University of Korea, Seoul 06591, Republic of Korea; 3 Department of Obstetrics and Gynecology, Eunpyeong St. Mary’s Hospital, College of Medicine, The Catholic University of Korea, Seoul 06591, Republic of Korea; 4 Department of Obstetrics and Gynecology, Bucheon St. Mary’s Hospital, College of Medicine, The Catholic University of Korea, Seoul 06591, Republic of Korea; 5 Department of Obstetrics and Gynecology, St. Vincent’s Hospital, College of Medicine, The Catholic University of Korea, Seoul 06591, Republic of Korea; 6 Department of Obstetrics and Gynecology, Uijeongbu St. Mary’s Hospital, College of Medicine, The Catholic University of Korea, Seoul 06591, Republic of Korea; 7 College of Pharmacy, Sookmyung Women’s University, Seoul 04310, Republic of Korea; 8 Goodbeing Center Co., Ltd., Seoul 04310, Republic of Korea

**Keywords:** endocrine disruptors, bisphenol-A, monoethyl phthalates, perfluorooctanoic acid, gestational diabetes mellitus

## Abstract

Gestational diabetes mellitus (GDM) requires lifestyle changes that may alter exposure to endocrine-disrupting chemicals (EDCs). This study aimed to assess maternal and fetal exposure to EDCs—including bisphenol-A (BPA), monoethyl phthalate (MEP), and perfluorooctanoic acid (PFOA)—during the COVID-19 pandemic and to evaluate their association with fetal birthweight. Maternal urine (second and third trimester) and paired cord blood samples were analyzed from 58 GDM and 118 non-GDM pregnancies using UPLC-MS/MS. Significant correlations were found between maternal urine and cord blood levels of BPA and MEP. Cord blood BPA levels were significantly lower in GDM mothers (0.35 vs. 0.72 μg/L, *p* < 0.05), suggesting reduced exposure due to dietary interventions. However, maternal urinary BPA levels in GDM pregnancies were positively associated with fetal birthweight (β = 2.69, *p* < 0.05), indicating increased susceptibility to obesogenic effects. PFOA was present in all cord blood but only 41% of maternal urine samples. These findings underscore the dual impact of GDM-related lifestyle changes: reduced EDC transfer to the fetus, yet persistent metabolic vulnerability.

## 1. Introduction

Gestational diabetes mellitus (GDM) occurs in approximately 10% of pregnancies, and its global prevalence is increasing substantially [1]. GDM shares similarities with type 2 diabetes mellitus in pathophysiology and is believed to be caused by increased insulin resistance during pregnancy. Although genetic factors play a significant role in the etiology of GDM, diet and lifestyle habits are also well-documented influences [2,3].

Endocrine disrupting chemicals (EDCs) act as obesogens, potentially inducing or exacerbating conditions such as diabetes mellitus, cardiovascular disease, cancer, and immune disorders [4,5]. Phenols, phthalates, and perfluoroalkyl substances (PFASs), as representative EDCs, have been implicated in insulin resistance by deregulating islet b-cell function, causing hormonal and epigenetic alterations that lead to an increase in blood glucose [6,7]. EDCs can affect glucose metabolism not only in the mother, but also in the fetus, potentially affecting fetal weight and increasing the long-term risks of obesity and endocrine disorders [8,9]. Especially considering that GDM is a risk factor for fetal macrosomia, exposing patients with GDM to EDCs might intensify the risk of macrosomia or fetal growth restriction compared to non-GDM pregnancies. Therefore, it is crucial to evaluate maternal and fetal exposure to EDCs in pregnancies affected by GDM.

Existing studies have primarily focused on the correlation between GDM and EDCs, emphasizing the casual effect of EDC exposure on the risks of GDM [10,11,12]. However, previous research has often lacked sufficient consideration of the significant changes in lifestyle patterns that generally occur after a GDM diagnosis to enable blood glucose control [12,13,14]. Conflicting results and interpretational challenges have arisen from differences in analysis timing, methodological approaches, and predominant evaluation of maternal exposures without assessment of fetal exposures.

Changes in lifestyle due to the coronavirus disease (COVID-19) pandemic, including an excessive increase in plastic use, have heightened concerns about exposure to environmental toxins [15]. Therefore, during this period, assessing exposure to EDCs has emerged as a significant challenge.

In our previous research, we explored the relationship between third-trimester maternal and fetal levels of EDCs and asymmetric fetal growth restriction, emphasizing the potential impact of bisphenol-A (BPA) on placental function and fetal development [16]. Furthermore, through experimental studies using trophoblast cell lines, we demonstrated that polycyclic aromatic hydrocarbons (PAHs) and BPA substitutes significantly impair placental cell viability and induce necrosis, providing biological plausibility for EDC-induced placental dysfunction [17].

Therefore, the present study has three primary aims. The first is to assess EDC exposure in mothers and fetuses during the COVID-19 pandemic. The second is to examine differences in EDC exposures during the mid- and late trimesters of pregnancy and in fetuses according to the presence of GDM. The third is to investigate the effects of EDC exposure on the neonatal birthweights in pregnancies complicated by GDM.

## 2. Results

### 2.1. Baseline Characteristics of Study Population

Table 1 provides a description of the baseline characteristics of the total study population and between the GDM and non-GDM groups. In the second trimester, 22 participants with GDM and 40 without GDM enrolled and provided urine samples. One participant was lost to follow-up, making it impossible to collect urine and cord blood samples in the third trimester. In the third trimester, an additional 36 participants with GDM and 78 without GDM were included, resulting in an analysis of 58 pregnant patients with GDM and 118 without GDM. The GDM group had a higher prevalence of family history of diabetes, of history of previous GDM, and of pre-pregnancy BMI than the non-GDM group. The prevalence of alcohol consumption history and weight gain during pregnancy was lower in the GDM group. The rate of pregnancy-associated hypertensive disorders was higher in the GDM group. Delivery occurred slightly earlier in the GDM group, and neonates born to mothers with GDM had a higher NICU admission rate than those born to mothers in the non-GDM group. Neither birthweight nor proportion of LGA differed significantly between the groups.

### 2.2. Maternal and Fetal EDC Concentrations Measured During the COVID-19 Pandemic

During our study period, from October 2021 to October 2023, which coincided with part of the COVID-19 pandemic, we investigated BPA, MEP, and PFOA exposures (Table 2). BPA and MEP were detected in 70–90% of maternal samples and about 80% of fetal samples. PFOA was not detected in any of the mid-pregnancy samples, but it was detected in 41% of maternal urine samples from late pregnancy and in 100% of cord blood samples. The geometric mean value of BPA in late pregnancy was 1.13 μg/g creatinine, which was higher than the 0.44 μg/g creatinine observed in mid-pregnancy. In contrast, the geometric mean value of MEP in late pregnancy was 12.23 μg/g creatinine, which was lower than the 17.98 μg/g creatinine observed in mid-pregnancy. Both BPA and MEP showed higher concentrations in urine than in cord blood, whereas PFOA displayed higher concentration in cord blood than maternal urine.

### 2.3. Maternal and Fetal BPA and MEP Levels Were Significantly Correlated

**Figure 1:**
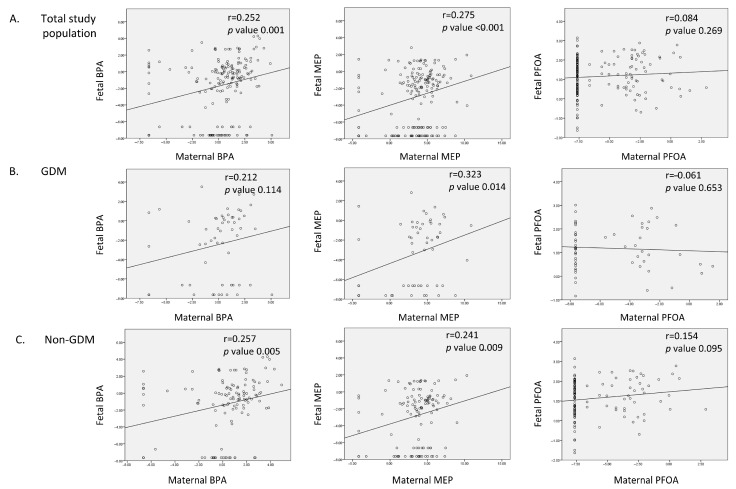
Pearson correlations between log-transformed values of EDCs in maternal urine before delivery and fetal cord blood. (**A**) In the entire study population, BPA and MEP exhibited a significant correlation between maternal urine and cord blood. (**B**) Among GDM patients, MEP showed a significant correlation between maternal urine and cord blood. (**C**) In non-GDM patients, BPA and MEP demonstrated a significant correlation between maternal urine and cord blood.

Figure 1 shows the correlations between EDC levels in maternal urine and those in cord blood. In the total study population, BPA and MEP showed a significant correlation between maternal urine in late pregnancy and cord blood (BPA, r = 0.252, *p* = 0.001; MEP, r = 0.275, *p* < 0.001). However, PFOA did not demonstrate such a correlation (r = 0.084, *p* = 0.269). To investigate whether those correlations were evident within each group, we stratified the population based on GDM status and conducted the correlation analyses again. In the GDM group, only MEP displayed a significant correlation between maternal urine and cord blood (r = 0.323, *p* = 0.014). BPA showed a correlation coefficient of approximately 0.212 of correlation coefficient but was not statistically significant. In the non-GDM group, both BPA and MEP demonstrated significant correlations between maternal urine and cord blood (BPA, r = 0.257, *p* = 0.005; MEP, r = 0.241, *p* = 0.009). We found no significant correlations for BPA and MEP levels between the mid-pregnancy and late-pregnancy urine samples (Figure S2).

### 2.4. Cord Blood BPA Levels Were Lower in GDM Groups than in Non-GDM Groups

Table 3 presents the analysis of differences in EDC concentrations between the GDM and non-GDM groups. The BPA and MEP levels in mid-pregnancy urine showed no significant differences between the GDM and non-GDM groups. The BPA concentration in late-pregnancy urine was lower in the GDM group, though the difference was not statistically significant, and MEP and PFOA exhibited no significant differences between the groups. In cord blood, BPA levels were significantly lower in the GDM group (cord blood BPA, 0.347 μg/L vs. 0.722 μg/L, *p* = 0.037), as were MEP and PFOA levels, though those differences were not significant. When we analyzed lifestyle factors between the GDM and non-GDM groups, microwave usage, canned food consumption, and frequency of French fry consumption were significantly lower in the GDM group (Table S1). When we analyzed the associations between EDC levels and lifestyle factors, we found that urinary BPA level was positively associated with smoking, type of drinking water, and use of plastic wrap. In addition, urinary MEP level was positively associated with personal computer use and frequent consumption of hamburgers. Urinary PFOA level was positively associated with frequent consumption of popcorn and nachos and tablet personal computer use (Table S2). Popcorn and nachos are often packaged in materials containing perfluorinated compounds for grease resistance, while tablet personal computers might involve indirect exposure via dust or contact with treated surfaces. These findings highlight the importance of identifying and mitigating specific lifestyle-related sources of EDC exposure during pregnancy to minimize potential health risks for both mothers and fetuses.

### 2.5. Maternal BPA Levels Were Positively Associated with Birthweight in GDM Groups

We examined the relationships between birthweight and EDCs through a linear regression analysis (Table 4 and Table S3). In the total study population, we did not observe a significant linear association between the analyzed EDCs and birthweight. However, when we stratified the groups into GDM and non-GDM, a significant positive linear relationship was observed between birthweight and third-trimester maternal BPA in the GDM group, a relationship that remained significant even after adjusting for covariates (β = 2.698, *p* < 0.05).

## 3. Discussion

### 3.1. Main Finding

We performed biological monitoring of BPA, MEP, and PFOA in pregnancy during the COVID-19 pandemic era and considered GDM. Specifically, we investigated the effects of those EDCs on maternal–fetal correlations and fetal weight according to GDM status. We found significant correlations between maternal urine and cord blood concentrations of BPA and MEP in the total study population regardless of GDM. We observed lower BPA exposure in cord blood among participants with GDM, who were following a prescribed diet, than in those without GDM; however, we also found a positive association between cord blood BPA level and fetal weight in GDM patients.

### 3.2. EDC Exposure Levels During the COVID-19 Pandemic

The increased use of personal protective equipment (PPE) and changes in indoor living patterns during the COVID-19 pandemic elevated the use of plastics [15]. Organic compounds, microplastics, and phthalates were all detected in PPE [18,19], which has contributed to growing concerns about the risks of exposure to phenols, phthalates, and PFASs during the COVID-19 pandemic period. Therefore, we investigated whether exposure to EDCs increased during the COVID-19 pandemic by comparing observed exposure levels with those in the literature. Because EDC exposure levels can vary by race and region, we compared our findings with others conducted in the Republic of Korea, and they reported exposure levels similar to ours [20,21,22,23]. Our findings did not align with our initial expectation of a significant increase in exposure to BPA and MEP during the COVID-19 pandemic. Phenols and phthalates in children’s products have been regulated in the Republic of Korea since 2015, with chemical substance registration and evaluation implemented since 2018. Those regulations might be why exposure to plastic-related EDCs has not increased despite an increase in plastic use.

Although no previous literature reported maternal PFOA levels in Korea, some results from maternal blood and cord blood have been reported. According to previous research, the mean PFOA concentration in cord blood was 2.73 µg/L in 2011 [24] and 1.41 µg/L in 2013–2015 [25]. Here, we report a median PFOA concentration in cord blood of 2.32 μg/L (Table 3), between the previous data points. Given the high risk of accumulation as a persistent organic pollutant, continued assessment of maternal and cord blood PFOA levels is warranted. The characteristics of this study, i.e., the pandemic environment and a focus on GDM, might have affected our results, indicating that EDC concentrations might vary due to differences in time and maternal characteristics.

### 3.3. Effects of GDM on the Transfer of EDC from Mothers to Babies

The passage of EDCs through the placenta has been documented in previous ex vivo placental transfusion models [26,27]. In human studies, BPA, phthalates, and PFOA have all been reported to exhibit significant correlations between maternal and fetal samples, confirming the well-established phenomenon that these substances pass through the placenta [24,28,29,30]. Similarly, we observed significant correlations between maternal urine and cord blood concentrations for BPA and MEP. Although PFOA did not show such a correlation, this outcome is likely due to its poor excretion in maternal urine.

The process of fetal EDC exposure through the placenta involves passive transfusion or transfer through transporters [31]. GDM causes the placenta to exhibit distinct characteristics, including fibrinoid necrosis, vascular lesions, and aberrant vascularization [32,33]. Alterations in nutrient transporters [33,34] and reduced expression of the P-glycoprotein efflux transporter, a major source of EDC removal, have also been found in GDM placentas [35]. Therefore, we initially hypothesized that GDM and non-GDM mothers would have differences in EDC levels. However, our results showed similar patterns in GDM and non-GDM mothers (Figure 1). Thus, GDM might negligibly affect the transmission of EDCs through the placenta.

### 3.4. Maternal and Fetal EDC Exposures According to GDM

Phenolic EDCs, phthalates, and PFAS are representative substances in type 2 diabetes and impaired glucose metabolism [36]. BPA and MEP have been implicated in impaired glucose metabolism during pregnancy, and several studies have reported associations with PFOA as well [10,11,12,13,14]. However, most studies have focused on causal effects during early pregnancy or exposure levels at a single time point, even in late pregnancy. Although GDM is typically diagnosed in the second trimester, making it a significant evaluation variable for maternal exposure levels before and after diagnosis and for fetal exposure, few studies have focused on the time aspect of GDM. Therefore, we investigated maternal exposure levels before and after GDM diagnosis and for fetal exposure. Interestingly, BPA levels in fetal cord blood were lower in the GDM group than the non-GDM group (Table 3). Considering the well-controlled diets of GDM mothers, this finding might reflect some intervention effects associated with the pandemic era rather than GDM status, though Wang et al. reported a negative association between BPA level and GDM risk. They collected maternal urine samples in the third trimester, similarly to our study [14]. On the other hand, some previous studies reported an association between BPA and insulin resistance, showing a discrepancy with our results [37,38]. Those differences could be attributed to the timing of BPA measurement in our study, which occurred after diagnosis of GDM and implementation of lifestyle modifications.

Our lifestyle survey indicated lower rates of alcohol consumption, microwave use, canned food consumption, and French fry intake among GDM patients compared to non-GDM patients. Additionally, smoking, plastic wrap use, and type of drinking water were associated with BPA level, which suggests a correlation between lifestyle habits and EDCs. We did identify some specific lifestyle factors associated with lower BPA levels in the GDM group compared to the non-GDM group (Supplementary Table S1). Particularly, the overall healthier dietary habits and lower gestational weight gain observed in GDM patients, compared with the non-GDM group, might have contributed to these findings. Other research has also highlighted the effects of lifestyle modifications on reducing exposure to EDCs [39,40]. Thus, we expect that the dietary modifications associated with a GDM diagnosis might affect BPA exposure.

### 3.5. The Effects of BPA on Fetal Weight in Mothers with GDM

A wealth of research shows the effects of EDCs on fetal weight, and it has particularly focused on prenatal exposure to BPA. Our previous clinical study demonstrated a significant association between third-trimester maternal and fetal BPA levels and asymmetric fetal growth restriction, particularly characterized by increased head circumference to abdominal circumference (HC/AC) ratios [16]. These findings suggested that BPA exposure may impair placental function and result in fetal brain-sparing physiology, consistent with a pattern of placental insufficiency. Complementing this, our in vitro experiments using placental trophoblast cell lines showed that PAHs and BPA substitutes significantly reduced cell viability and induced necrosis, indicating that EDCs can exert direct toxic effects on placental tissue [17]. Together, these results support a dual-mechanism model in which BPA may influence fetal growth via two distinct pathways: inducing placental dysfunction leading to growth restriction in some contexts and promoting fetal overgrowth via obesogenic effects in others.

This context-dependent response may help explain the heterogeneous findings in the literature regarding EDC exposure and fetal growth, and highlights the importance of considering maternal metabolic status, timing of exposure, and placental function in interpreting the effects of EDCs on perinatal outcomes.

While previous studies have reported conflicting associations between prenatal BPA exposure and fetal weight, a consistent link has been suggested between BPA and childhood obesity and metabolic disorders [41]. Additionally, recent studies have reported that epigenetic changes related to obesity are influenced by BPA [42]. In our study, the study population as a whole, regardless of GDM, did not show a significant association between BPA levels and fetal birthweight. However, BPA levels in the GDM group were positively associated with fetal birthweight (Table 3). Thus, the obesogenic effects of BPA might be more pronounced in GDM mothers than non-GDM mothers. The GDM mothers already experience elevated insulin resistance, leading to hyperinsulinemia and altered lipid metabolism, which contributes to fetal macrosomia [43]. Thus, BPA might further activate those obesogenic effects in GDM mothers, potentially exacerbating the risk of fetal macrosomia.

Further research could clarify the mechanisms through which BPA has significant obesogenic effects on the fetuses of GDM mothers and determine whether specific conditions, such as fetal sex, contribute to the obesogenic effects of BPA.

### 3.6. Strengths and Limitations

Our study was conducted prospectively and assessed repetitive exposure through urinalyses in the second and third trimesters. To prevent sample degradation and contamination, we used shading and dedicated sample containers. Additionally, sample and patient management was conducted in a single hospital, enabling consistent treatment and accurate record collection. To the best of our knowledge, this study is the first investigation of EDC exposures among pregnant Koreans during the COVID-19 pandemic. It is also the first to evaluate the correlation between EDC exposures in mothers and fetuses based on GDM status.

## 4. Materials and Methods

### 4.1. Study Population and Design

This study was conducted as a prospective cohort study to evaluate maternal and fetal exposure to EDCs during the COVID-19 pandemic. We enrolled pregnant patients who visited Seoul St. Mary’s Hospital from October 2021 to October 2023. For patients who received prenatal care during the second trimester, urine samples were collected during the second trimester and before delivery. For those who visited only late in pregnancy, only pre-delivery urine samples were collected. Cord blood samples were obtained after delivery. Participants were surveyed about their socioeconomic status, environmental exposures, dietary habits, and electronic device usage.

Endocrine disrupting chemicals (EDCs) such as BPA, phthalates, and PFAS were quantified in each biospecimen using ultra–performance liquid chromatography–tandem mass spectrometry (UPLC-MS/MS). We investigated the effects of EDC levels on maternal and fetal health, particularly obesity-related biomarkers, according to maternal GDM status. We conducted this study with approval of the Institutional Review Board of The Catholic Medical Center in Republic of Korea (no. XC21ONDI0125), after obtaining informed consent from all participants.

### 4.2. Quantification of BPA, Monoethyl Phthalate (MEP), and Perfluorooctanoic Acid (PFOA)

Maternal urine and cord blood samples were shielded from light and transported to the laboratory in BPA-free containers, where they were stored at −80 °C until analyses.

We prepared urine and cord blood to analyze BPA and MEP following the methods of Yi et al. [44] and Hong et al. [16], respectively, with minor modifications. That is, the free and total forms of BPA and MEP were separated with and without enzyme hydrolyses. The prepared samples were subjected to UPLC-MS/MS using an Agilent 1260 Infinity UPLC system (Agilent Technologies, Santa Clara, CA, USA) and an Agilent Triple Quadrupole 6460 system with a specialized electrospray ionization interface.

For the PFOA analyses, we prepared urine and cord blood according to the methods of Worley et al. [45] and Liu et al. [46], respectively, with minor modifications. Briefly, enzyme hydrolysis and sonication were used to remove the urine or blood matrix. The prepared samples were subjected to UPLC-MS/MS on a Thermo Fisher Scientific Ultimate UPLC system (Thermo Fisher Scientific, Waltham, MA, USA) and a Bruker EVOQ Qube LC-Triple Quadrupole (Bruker Corporation, Billerica, MA, USA) specifically designed to prevent fluoride contamination.

The limits of detection (LODs) for BPA, MEP, and PFOA were 0.1, 0.1, and 0.01 µg/L, respectively. To address undetected EDC levels, a value of one-half of the minimum detected EDC level was used for statistical analysis, following the approach outlined by Yang et al. [47]. Urine dilutions were adjusted by dividing molecule levels by creatinine (Cr) level, and urine was analyzed using an automatic biomedical analyzer (HITACHI 7020; Hitachi, Ltd., Tokyo, Japan). A detailed description of the LC/MS/MS conditions and calibration curves employed are described in the Supplementary Materials.

### 4.3. GDM Definition and Management

GDM was diagnosed during the second trimester using a two-step approach: a 50 g screening oral glucose challenge test (OGTT) followed by a diagnostic 100 g OGTT. If the serum glucose value was 140 mg/dL or higher in the 50 g OGTT, the 100 g OGTT was conducted. The GDM diagnosis was confirmed if two or more of the following values exceeded the Carpenter and Coustan thresholds: 95 mg/dL for fasting glucose, 180 mg/dL for 1 h glucose, 155 mg/dL for 2 h glucose, and >140 mg/dL for 3 h glucose [48]. Patients diagnosed with GDM were promptly referred to an endocrinologist for dietary and exercise education. The goal of the recommended lifestyle modifications was to maintain normal blood glucose level, prevent maternal obesity, and reduce adverse outcomes for infants, including those born overweight. The dietary education involved calculating the total caloric intake based on factors such as maternal weight and gestational weeks. It was recommended that participants divide this intake into three meals and three snacks, with a suggested distribution of carbohydrates, proteins, and fats of 50%, 20%, and 30%, respectively.

Birthweight was evaluated by calculating percentiles using the Korean birth weight curves and lambda mu sigma parameters presented by Lim et al., which are stratified by gestational age and sex [49]. A birthweight percentile of 90 or higher was defined as large for gestational age (LGA).

### 4.4. Covariates

We collected maternal baseline characteristics of age, parity, pre-pregnancy body mass index (BMI), pre-pregnancy smoking, pre-pregnancy alcohol consumption, underlying medical conditions, and pregnancy-related conditions. Additionally, we gathered data on gestational age at delivery, mode of delivery, birthweight, and neonatal intensive care unit (NICU) admission. We established potential confounders of maternal age, pre-pregnancy BMI, pre-pregnancy smoking, pre-pregnancy alcohol consumption, pregnancy-associated hypertension, GDM, fetal sex, and gestational age and evaluated the effects of EDCs on birthweight using DAGitty to create a directed acyclic graph (Figure S1).

### 4.5. Statistical Analysis

We used Student’s *t*-test or the Mann–Whitney U test to compare continuous variables and the Chi-squared test or Fisher’s exact test to compare categorical variables. Given that EDCs did not follow a normal distribution, we conducted the analysis using log-2-transformed values. To assess the correlations between EDCs found in maternal urine and those in cord blood samples, we calculated Pearson correlation coefficients. To examine the linear association between birthweight and EDC exposure, both univariable and multivariable linear regression analyses were performed. For the multivariable analysis, we constructed a directed acyclic graph using DAGitty and selected covariates for adjustment. Among the selected covariates, gestational age and fetal sex had been adjusted when calculating birthweight percentiles and were excluded from the multivariable analysis.

In the analyses of environmental factors and dietary habits between the GDM and non-GDM groups, we used linear-by-linear association to examine differences based on exposure levels. Student’s *t*-test or analysis of variance (ANOVA) was used to analyze differences in EDC levels based on socioeconomic status, environmental factors, and lifestyle habits. Spearman’s correlation coefficients were calculated for correlations between EDC levels and ordinal data about environmental and lifestyle factors. We considered *p*-values less than 0.05 to be statistically significant. The analysis was conducted using IBM SPSS Statistics V 25.0 (IBM Corp., Armonk, NY, USA) and R version 4.2.1 (http://www.r-project.org (accessed on 28 April 2025); R Foundation for Statistical Computing, Vienna, Austria).

## 5. Conclusions

During the COVID-19 pandemic, exposure to BPA was low in GDM mothers following well-controlled diets. However, the GDM group showed a positive association between maternal BPA exposure and fetal weight, which implies that babies whose mothers had GDM might be highly susceptible to obesity through their mothers’ exposure to EDCs. This study provides clinically relevant insights by highlighting how maternal metabolic conditions, such as GDM, modulate the impact of environmental exposures on fetal growth. Our findings emphasize the potential benefits of lifestyle modifications in reducing EDC exposure and support the need for targeted strategies in prenatal care to mitigate environmental risks and improve maternal–fetal health outcomes.

## Figures and Tables

**Table 1:** Baseline characteristics of the total study population, GDM, and non-GDM groups.

	Total (*n* = 176)	GDM (*n* = 58)	Non-GDM (*n* = 118)	*p*-Value
Samples				-
2nd trimester maternal urine	62/176 (35.2%)	22/62 (35.5%)	40/62 (64.5%)	
3rd trimester maternal urine	175/176 (99.4%)	57/175 (32.6%)	118/175 (67.4%)	
Fetal cord blood	175/176 (99.4%)	57/175 (32.6%)	118/175 (67.4%)	
Maternal characteristics				
Maternal age (years)	35 (32, 38)	36 (32, 39)	34 (31, 37)	0.128
Nulliparity	105 (59.7%)	35 (60.3%)	70 (59.3%)	0.897
First-degree relative with diabetes	51 (29.0%)	24 (41.4%)	27 (22.9%)	0.011
Previous GDM	14 (8.0%)	13 (22.4%)	1 (0.8%)	<0.001
Pre-pregnancy BMI (kg/m^2^)	21.2 (19.5, 23.7)	23.5 (20.1, 27.1)	20.7 (19.4, 22.68)	<0.001
Pre-pregnancy smoking	11 (6.3%)	3 (5.2%)	8 (6.8%)	1.000
Pre-pregnancy alcohol consumption	112 (63.6%)	28 (48.3%)	84 (71.2%)	0.003
Gestational weight gain (kg)	11.0 (8.0, 14.1)	9.0 (5.2, 11.6)	12.0 (8.9, 15.0)	<0.001
Pregnancy-associated hypertension	9 (5.1%)	8 (13.8%)	1 (0.8%)	0.001
Delivery outcomes				
GA at delivery (weeks)	38.6 (37.9, 39.3)	38.1 (37.3, 38.7)	38.9 (38.1, 39.7)	0.003
Cesarean delivery	100/175 (57.1%)	38/57 (66.7%)	62/118 (52.5%)	0.077
NICU admission ^1^	23/172 (13.1%)	13/55 (23.6%)	10/117 (8.5%)	0.007
Birthweight (kg) ^1^	3.13 (2.86, 3.34)	3.15 (2.84, 3.34)	3.11 (2.86, 3.34)	0.846
LGA ^1^	14/172 (8.1%)	6/55 (10.9%)	8/117 (6.8%)	0.380

Data are presented as median and interquartile range for continuous variables and as number and percentage for categorical variables. ^1^ Three participants were excluded due to unavailable data. BMI—body mass index; GA—gestational age; NICU—neonatal intensive care unit.

**Table 2:** Distributions of bisphenol-A, monoethyl phthalate, and perfluorooctanoic acid in maternal urine and fetal cord blood.

					Percentile					
EDC	Time Point/ Sample	N(%) > LOD	GM	Min	10th	25th	50th	75th	90th	Max
BPA	2nd trimester urine (μg/g cre) ^1^	46/62 (74.2)	0.448	ND	ND	ND	0.623	1.999	4.485	10.905
	3rd trimester urine (μg/g cre) ^2^	161/175 (92.0)	1.134	ND	0.070	0.680	1.611	3.650	8.202	33.918
	Fetal cord blood (μg/L) ^2^	144/175 (82.3)	0.311	ND	ND	0.100	0.610	1.490	5.900	19.679
MEP	2nd trimester urine (μg/g cre) ^1^	53/62 (85.5)	17.986	ND	ND	7.369	16.363	77.237	357.708	8450.644
	3rd trimester urine (μg/g cre) ^2^	161/175 (92.0)	12.230	ND	0.662	6.610	16.458	43.290	102.673	5745.223
	Fetal cord blood (μg/L) ^2^	139/175 (79.4)	0.126	ND	ND	0.010	0.290	0.700	2.014	31.150
PFOA	2nd trimester urine (μg/g cre) ^1^	0/62 (0.0)	ND	ND	ND	ND	ND	ND	ND	ND
	3rd trimester urine (μg/g cre) ^2^	72/175 (41.1)	0.019	ND	ND	ND	ND	0.110	0.240	7.450
	Fetal cord blood (μg/L) ^2^	175/175 (100)	2.229	0.321	0.950	1.441	2.305	3.676	4.900	8.827

^1^ Results are reported for those who enrolled in the second trimester: GDM (22 cases) and non-GDM (40 cases). ^2^ Results are reported for those who enrolled in the second and third trimesters: GDM (58 cases) and non-GDM (118 cases). One person enrolled in the second trimester was lost to follow up. LOD—limit of detection; GM—geometric mean; BPA—bisphenol-A; MEP—monoethyl phthalate; PFOA—perfluorooctanoic acid.

**Table 3:** Differences in EDC concentrations between the GDM and non-GDM groups.

Time Point	EDC	Sample	GDM (*n* = 22)	Non-GDM (*n* = 40)	*p* Value
**Mid-pregnancy**	BPA	Maternal urine (μg/g cre)	0.714 (0.320–2.158)	0.516 (0.028–1.480)	0.273
	MEP	Maternal urine (μg/g cre)	13.789 (5.879–66.611)	16.789 (8.082–77.412)	0.930
**Time Point**	**EDC**	**Sample**	**GDM** **(*n* = 57)**	**Non-GDM** **(*n* = 118)**	***p* Value**
**At delivery**	BPA	Maternal urine (μg/g cre)	1.290 (0.428–3.114)	1.757 (0.796–3.807)	0.169
		Fetal cord blood (μg/L)	0.347 (0.010–1.210)	0.722 (0.2536–1.655)	0.037
	MEP	Maternal urine (μg/g cre)	16.573 (5.790–50.778)	16.159 (6.835–40.902)	0.902
		Fetal cord blood (μg/L)	0.198 (0.010–0.760)	0.320 (0.010–0.681)	0.324
	PFOA	Maternal urine (μg/g cre)	0.005 (0.005–0.115)	0.005 (0.005–0.080)	0.539
		Fetal cord blood (μg/L)	2.220 (1.44–4.070)	2.429 (1.470–3.693)	0.942

Data are presented as median and interquartile range. *p*-values were calculated using Student’s *t*-test analyses of log-transformed values.

**Table 4:** Linear associations between EDCs and birthweight according to the presence or absence of GDM.

EDC	Time Point/Sample	Total	GDM	Non-GDM
		β (95% CI) ^1^	β (95% CI) ^2^	β (95% CI) ^2^
BPA	2nd trimester urine	−0.451 (−2.317, 1.416)	2.242 (−1.107, 5.591)	−0.996 (−3.419, 1.426)
	3rd trimester urine	0.396 (−1.788, 2.579)	2.698 (0.269, 5.127)	−0.879 (−2.982, 1.223)
	Cord blood	−0.326 (−1.615, 0.963)	0.340 (−1.589, 2.269)	−1.003 (−2.657, 0.652)
MEP	2nd trimester urine	0.426 (−1.716, 2.569)	0.121 (−3.882, 4.125)	0.148 (−2.761, 3.058)
	3rd trimester urine	0.395 (−0.979, 1.768)	0.062 (−1.711, 1.834)	0.872 (−1.083, 2.826)
	Cord blood	0.299 (−1.036, 1.633)	1.224 (−0.745, 3.193)	−0.264 (−1.991, 1.463)
PFOA	3rd trimester urine	−0.326 (−1.967, 1.315)	0.816 (−1.786, 3.417)	−1.268 (−3.420, 0.884)
	Cord blood	0.866 (−3.954, 5.686)	−5.248 (−12.322, 1.826)	3.610 (−2.521, 9.742)

^1^ Adjusted for maternal age, pre-pregnancy body mass index, gestational diabetes mellitus, pregnancy associated hypertension, smoking, and alcohol consumption. ^2^ Adjusted for maternal age, pre-pregnancy body mass index, pregnancy associated hypertension, smoking, and alcohol consumption.

## Data Availability

The datasets of the current study are available from the corresponding author on reasonable request.

## Supplementary Materials

The supporting information can be downloaded at: https://www.mdpi.com/article/10.3390/ijms26094226/s1.
