# Supplementary material for: Effects of Endocrine Disrupting Chemicals on Fetal Weight: Exposure Monitoring Among Mothers with Gestational Diabetes Mellitus and Their Fetuses
Source: Int J Mol Sci. 2025 Apr 29;26(9):4226. doi: 10.3390/ijms26094226 (PMC12071538; doi:10.3390/ijms26094226)
Supplement: Supplementary file 1 [file ijms-26-04226-s001.zip › ijms-3537503-SI.pdf]

Table S1. Differences in socioeconomic status, environmental factors, and lifestyle habits between GDM and Non-GDM Groups

		**GDM (n=45)**	**Non GDM (n=107)**	***p* value**
Education level	College graduate	34/45 (75.6%)	70/107 (65.4%)	0.220
	Above college	11/45 (24.4%)	37/107 (34.6%)	
Household income (1,000 KRW)	<4,000	8/44 (18.2%)	18/107 (16.8%)	0.841
	≥ 4,000	36/44 (81.8%)	89/107 (83.2%)	
Pre-pregnancy smoking (including passive smoking)	Yes	6/45 (13.3%)	22/107 (20.6%)	0.294
	No	39/45 (86.7%)	85/107 (79.4%)	
Air pollution level	Minimal	11/45 (24.4%)	14/105 (13.3%)	0.129
	Moderate	23/45 (51.1%)	58/105 (55.2%)	
	Severe	11/45 (24.4%)	33/105 (31.4%)	
Housing type	Single family house	6/44 (13.6%)	21/105 (20.0%)	0.358
	Apartment	38/44 (86.4%)	84/105 (80.0%)	
New house status	Yes	9/44 (20.5%)	17/105 (16.2%)	0.532
	No	35/44 (79.5%)	88/105 (83.8%)	
Beverage type	Purified water	29/45 (64.4%)	63/107 (58.9%)	0.420
	Bottled water	16/45 (35.6%)	42/107 (39.3%)	
	Tap water	0/45 (0.0%)	2/107 (1.9%)	
Kitchen detergent usage	Occasionally	3/45 (6.7%)	9/107 (8.4%)	0.637
	Moderately	6/45 (13.3%)	16/107 (15.0%)	
	Daily	36/45 (80.0%)	82/107 (76.6%)	
Frequency of facial makeup	1-3 time/month	20/45 (44.4%)	34/106 (32.1%)	0.097
	1-3 times/week	17/45 (37.8%)	42/106 (39.6%)	
	Daily	8/45 (17.8%)	30/106 (28.3%)	
Hygiene products	Cloth menstrual pads	2/45 (4.4%)	6/107 (5.6%)	0.834
	Disposable menstrual pads	40/45 (88.9%)	94/107 (87.9%)	

	Tampons	3/45 (6.7%)	7/107 (6.5%)	
Non-stick pan	Not used	5/45 (11.1%)	9/107 (8.4%)	0.521
	1-2 times/week	11/45 (24.4%)	40/107 (37.4%)	
	≥3 times/week	29/45 (64.4%)	58/107 (54.2%)	
Coating air fryer	Not used	33/45 (73.3%)	65/107 (60.7%)	0.139
	Used	12/45 (26.7%)	42/107 (39.3%)	
Microwave and plastic usage				
Microwave	1-3 times/ month	9/45 (20.0%)	11/106 (10.4%)	0.048
	1-3 times/ week	21/45 (46.7%)	44/106 (41.5%)	
	Daily	15/45 (33.3%)	51/106 (48.1%)	
Plastic tableware	Not used	17/45 (37.8%)	41/107 (38.3%)	0.714
	Occasionally	19/45 (42.2%)	49/107 (45.8%)	
	Daily used	9/45 (20.0%)	17/107 (15.9%)	
Plastic wrap	Not used	27/45 (60.0%)	67/107 (62.6%)	0.835
	Occasionally	18/45 (40.0%)	35/107 (32.7%)	
	Daily used	0/45 (0.0%)	5/107 (4.7%)	
Packaged Food for Microwave	Not used	15/45 (33.3%)	24/107 (22.4%)	0.355
	1-3 times/ month	16/45 (35.6%)	48/107 (44.9%)	
	≥ 1 times/ week	14/45 (31.1%)	35/107 (32.7%)	
Household items				
Air purifier	1-3 times/ month	12/44 (27.3%)	30/107 (28.0%)	0.924
	1-3 times/ week	12/44 (27.3%)	26/107 (24.3%)	
	Daily	20/44 (45.5%)	51/107 (47.7%)	
Humidifier	1-3 times/ month	22/43 (51.2%)	46/107 (43.0%)	0.232
	1-3 times/ week	10/43 (23.3%)	22/107 (20.6%)	
	Daily	11/43 (25.6%)	39/107 (36.4%)	

Air conditioner	1-3 times/ month	19/44 (43.2%)	38/106 (35.8%)	0.361
	1-3 times/ week	11/44 (25.0%)	27/106 (25.5%)	
	Daily	14/44 (31.8%)	41/106 (38.7%)	
Dehumidifier	1-3 times/ month	33/44 (75.0%)	91/107 (85.0%)	0.337
	1-3 times/ week	6/44 (13.6%)	5/107 (4.7%)	
	Daily	5/44 (11.4%)	11/107 (10.3%)	
Mosquito repellent	Rarely used	38/43 (88.4%)	84/106 (79.2%)	0.313
	1-3 times/month	1/43 (2.3%)	9/106 (8.5%)	
	≥1 times/week	4/43 (9.3%)	13/106 (12.3%)	
Odor neutralizer	Rarely used	28/42 (66.7%)	67/106 (63.2%)	0.183
	1-3 times/month	13/42 (31.0%)	22/106 (20.8%)	
	≥1 times/week	1/42 (2.4%)	17/106 (16.0%)	
Bathroom Disinfectant	Rarely used	10/44 (22.7%)	27/107 (25.2%)	0.941
	1-3 times/month	24/44 (54.5%)	52/107 (48.6%)	
	≥1 times/week	10/44 (22.7%)	28/107 (26.2%)	
Dietary Habits (consumption)				
Canned Tuna	1-3 times/month	37/44 (84.1%)	89/102 (87.3%)	0.610
	≥1 times/week	7 (15.9%)	13/102 (12.7%)	
Canned Food	0	15/45 (33.3%)	23/107 (21.5%)	0.052
	1-3 times/month	23/45 (51.1%)	54/107 (50.5%)	
	≥1 times/week	7/45 (15.6%)	30/107 (28.0%)	
Hamburger	0	10/45 (22.2%)	13/107 (12.1%)	0.229
	1-3 times/month	30/45 (66.7%)	81/107 (75.7%)	
	≥1 times/week	5/45 (11.1%)	13/107 (12.1%)	
French Fries	0	21/45 (46.7%)	19/107 (17.8%)	0.006
	1-3 times/month	20/45 (44.4%)	81/107 (75.7%)	

	≥1 times/week	4/45 (8.9%)	7/107 (6.5%)	
Pizza	0	10/45 (22.2%)	13/106 (12.3%)	0.219
	1-3 times/month	33/45 (73.3%)	89/106 (84.0%)	
	≥1 times/week	2/45 (4.4%)	4/106 (3.8%)	
Chicken	0	6/45 (13.3%)	10/105 (9.5%)	0.249
	1-3 times/month	37/45 (82.2%)	85/105 (81.0%)	
	≥1 times/week	2/45 (4.4%)	10/105 (9.5%)	
Food Delivery	0	2/45 (4.4%)	8/107 (7.5%)	0.620
	1-3 times/month	22/45 (48.9%)	40/107 (37.4%)	
	≥1 times/week	21/45 (46.7%)	59/107 (55.1%)	
Popcorn and Nacho	0	42/45 (93.3%)	90/107 (84.1%)	0.225
	1-3 times/month	2/45 (4.4%)	15/107 (14.0%)	
	≥1 times/week	1/45 (2.2%)	2/107 (1.9%)	
Electronic devices				
Mobile Phone	4-6 times/week	9/45 (20.0%)	12/107 (11.2%)	0.239
	<1 hour/day	20/45 (44.4%)	51/107 (47.7%)	
	≥1 hour/day	16/45 (35.6%)	44/107 (41.1%)	
TV	4-6 times/week	9/45 (20.0%)	17/107 (15.9%)	0.307
	<1 hour/day	11/45 (24.4%)	20/107 (18.7%)	
	≥1 hour/day	25/45 (55.6%)	70/107 (65.4%)	
PC	4-6 times/week	21/44 (47.7%)	43/106 (40.6%)	0.142
	<1 hour/day	8/44 (18.2%)	8/106 (7.5%)	
	≥1 hour/day	15/44 (34.1%)	55/106 (51.9%)	
Tablet PC	<1 hour/day	2/45 (4.4%)	11/107 (10.3%)	0.449
	≥1 hour/day	3/45 (6.7%)	9/107 (8.4%)	
	≥3 hour/day	40/45 (88.9%)	87/107 (81.3%)	

Statistical analysis was conducted using the Chi-square test or linear-by-linear association analysis. GDM, gestational diabetes mellitus.

Table S2 Differences and correlations in EDCs levels based on the socioeconomic status, environmental factors, and lifestyle habits of the study population.

		N	Maternal BPA (µg/g cre)	Fetal BPA (µg/L)	Maternal MEP (µg/g cre)	Fetal MEP (µg/L)	Maternal PFOA (µg/g cre)	Fetal PFOA (µg/L)
Education level	College graduate	104	1.631 (0.696, 3.437)	0.626 (0.085, 1.410)	14.600 (5.045, 44.745)	0.199 (0.010, 0.704)	0.005 (0.005, 0.065)	2.289 (1.440, 3.433)
	Above college	48	1.986 (0.850, 4.002)	0.921 (0.188, 2.162)	17.601 (9.065, 39.339)	0.445 (0.058, 0.830)	0.015 (0.005, 0.110)	2.531 (1.685, 4.260)
	p value[1]		0.790	0.575	0.502	0.130	0.109	0.082
Household income (1,000 KRW)	<4,000	26	1.426 (0.190, 2.750)	0.866 (0.009, 1.483)	14.133 (7.631, 29.713)	0.170 (0.005, 0.454)	0.005 (0.005, 0.243)	1.805 (1.069, 4.423)
	≥ 4,000	125	1.803 (0.800, 3.910)	0.590 (0.130, 1.752)	16.672 (6.320, 44.295)	0.320 (0.010, 0.760)	0.005 (0.005, 0.080)	2.440 (1.560, 3.642)
	p value[1]		0.119	0.618	0.866	0.065	0.127	0.185
Pre-pregnancy smoking	Yes	28	2.469 (1.250, 4.228)	**1.225 (0.583, 1.888)**	16.217 (7.576, 40.828)	0.541 (0.048, 1.853)	0.013 (0.005, 0.268)	1.910 (1.250, 3.590)
	No	124	1.631 (0.615, 3.645)	**0.565 (0.020, 1.614)**	14.797 (5.520, 43.223)	0.260 (0.010, 0.689)	0.005 (0.005, 0.776)	2.313 (1.482, 3.702)
	p value[1]		0.290	**0.028**	0.777	0.147	0.084	0.706
Air pollution level	Minimal	25	1.302 (0.625, 3.443)	0.545 (0.085, 1.880)	13.98 (4.718, 30.115)	0.198 (0.010, 0.450)	0.005 (0.005, 0.025)	2.32 (1.395, 3.400)
	Moderate	81	1.711 (0.645, 4.000)	0.64 (0.060, 1.543)	15.23 (6.765, 39.308)	0.35 (0.015, 0.763)	0.005 (0.005, 0.095)	2.262 (1.507, 3.815)
	Severe	44	1.755 (0.938, 3.394)	0.72 (0.176, 1.710)	19.441 (7.075, 47.864)	0.28 (0.010, 0.657)	0.005 (0.005, 0.110)	2.635 (1.498, 4.020)

	p value[2]		0.775	0.947	0.606	0.216	0.542	0.814
	SR		0.027	0.019	0.103	0.047	0.077	0.058
Housing type	Single family house	27	1.302 (0.680, 2.450)	0.722 (0.070, 1.490)	16.573 (7.571, 48.796)	0.26 (0.010, 0.739)	0.005 (0.005, 0.040)	2.509 (1.480, 3.450)
	Apartment	122	1.728 (0.724, 3.947)	0.62 (0.140, 1.648)	15.545 (6.465, 41.696)	0.285 (0.010, 0.698)	0.005 (0.005, 0.100)	2.395 (1.490, 3.728)
	p value[1]		0.919	0.880	0.406	0.632	0.621	0.922
New house status	Yes	26	1.7 (0.800, 3.780)	0.722 (0.100, 1.723)	14.894 (6.000, 43.29)	0.273 (0.010, 0.640)	0.005 (0.005, 0.080)	2.32 (1.490, 3.607)
	No	123	1.441 (0.140, 3.773)	0.61 (0.156, 1.494)	18.341 (9.984, 36.576)	0.366 (0.063, 1.252)	0.005 (0.005, 0.143)	2.975 (1.427, 4.208)
	p value[1]		0.123	0.733	0.992	0.235	0.439	0.684
Beverage type	Purified water	92	1.335 (0.638, 3.277)	0.585 (0.020, 1.471)	15.062 (6.008, 46.043)	0.27 (0.010, 0.657)	0.005 (0.005, 0.108)	2.462 (1.500, 3.988)
	Bottled water	58	2.36 (1.193, 4.346)	0.88 (0.372, 1.887)	16.8 (7.239, 41.714)	0.292 (0.010, 0.866)	0.005 (0.005, 0.055)	2.293 (1.431, 3.361)
	Tap water	2	5.411 (0.590, 3.524)	9.337 (0.005, 1.155)	21.514 (8.210, 25.260)	0.472 (0.350, 0.605)	0.735 (0.050, 0.080)	5.661 (4.500, 3.580)
	p value[2]		0.128	0.301	0.615	0.739	0.093	0.067
Kitchen detergent usage	Occasionally	12	2.516 (1.340, 6.189)	1.015 (0.566, 2.027)	10.998 (2.273, 17.976)	0.625 (0.074, 1.150)	**0.005 (0.005, 0.118)**	2.335 (1.504, 4.168)
	Moderately	22	2.175 (0.717, 4.343)	0.419 (0.010, 1.331)	19.221 (9.494, 54.123)	0.245 (0.010, 0.537)	**0.085 (0.005, 0.220)**	2.738 (2.127, 3.508)
	Daily	118	1.596 (0.675, 3.440)	0.681 (0.095, 1.570)	15.027 (6.835, 46.645)	0.282 (0.010, 0.713)	**0.005 (0.005, 0.050)**	2.313 (1.398, 3.702)

	p value[2]		0.412	0.111	0.421	0.667	**0.022**	0.394
	SR		-0.113	-0.015	0.039	-0.052	**-0.178**	-0.076
Frequency of facial makeup	1-3 time/month	54	1.615 (0.515, 2.863)	0.474 (0.010, 1.298)	14.133 (6.238, 33.645)	0.208 (0.010, 0.684)	0.005 (0.005, 0.093)	2.110 (1.235, 3.370)
	1-3 times/week	59	1.700 (0.680, 3.780)	0.830 (0.080, 2.049)	1800 (5.020, 53.627)	0.300 (0.005, 0.694)	0.005 (0.005, 0.110)	2.547 (1.490, 3.891)
	Daily	38	2.061 (0.965, 4.336)	0.91 (0.357, 1.683)	16.565 (9.421, 36.195)	0.37 (0.169, 1.462)	0.005 (0.005, 0.013)	2.497 (1.678, 4.095)
	p value[2]		0.443	0.153	0.807	0.104	0.350	0.125
	SR		0.103	0.144	0.059	0.138	-0.055	0.151
Hygiene products	Cloth menstrual pads	8	1.365 (0.225, 1.926)	0.766 (0.208, 1.687)	23.325 (3.893, 50.503)	0.008 (0.005, 0.788)	0.095 (0.005, 0.766)	1.430 (0.983, 2.288)
	Disposable menstrual pads	134	1.706 (0.729, 3.881)	0.631 (0.078, 1.683)	15.027 (6.023, 41.576)	0.285 (0.010, 0.694)	0.005 (0.005, 0.083)	2.544 (1.517, 3.936)
	Tampons	10	2.620 (1.234, 5.731)	0.978 (0.076, 1.880)	26.060 (7.603, 165.112)	0.565 (0.005, 2.405)	0.005 (0.005, 0.033)	1.850 (1.227, 2.542)
	p value[2]		0.390	0.970	0.375	0.198	0.060	0.018
Non-stick pan	Not used	14	**2.282 (1.005, 3.911)**	1.508 (0.518, 6.687)	16.565 (6.425, 52.103)	0.196 (0.005, 0.587)	0.005 (0.005, 0.080)	2.284 (1.365, 3.922)
	1-2 times/week	51	**2.320 (0.800, 6.132)**	0.640 (0.160, 1.723)	18.782 (2.670, 45.230)	0.320 (0.010, 0.760)	0.005 (0.005, 0.130)	2.380 (1.550, 2.980)
	≥3 times/week	87	**1.311 (0.680, 3.352)**	0.570 (0.050, 1.480)	14.384 (7.20, 41.130)	0.260 (0.010, 0.669)	0.005 (0.005, 0.070)	2.675 (1.450, 4.070)
	p value[2]		0.430	0.697	0.711	0.244	0.186	0.196
	SR		**-0.170**	-0.122	-0.021	-0.035	-0.034	0.082

Coating air fryer	Not used	98	1.752 (0.625, 3.740)	0.582 (0.078, 1.435)	14.437 (5.303, 41.576)	0.267 (0.010, 0.741)	0.005 (0.005, 0.110)	2.350 (1.448, 3.685)
	Used	54	1.661 (0.886, 3.949)	0.857 (0.189, 1.892)	16.516 (7.586, 48.323)	0.345 (0.005, 0.703)	0.005 (0.005, 0.050)	2.514 (1.525, 4.058)
	p value[1]		0.287	0.635	0.288	0.927	0.549	0.905
	SR		-0.027	0.065	0.072	0.003	-0.028	0.040
Microwave and plastic usage								
Microwave	1-3 times/ month	20	2.352 (0.547, 4.195)	0.820 (0.247, 2.005)	22.475 (9.439, 42.512)	0.01 (0.010, 0.592)	0.005 (0.005, 0.148)	2.485 (1.529, 4.498)
	1-3 times/ week	65	1.942 (0.800, 3.967)	0.935 (0.354, 1.915)	12.691 (5.125, 46.750)	0.38 (0.010, 0.790)	0.005 (0.005, 0.065)	2.250 (1.350, 3.355)
	Daily	66	1.596 (0.653, 3.661)	0.572 (0.009, 1.528)	16.516 (7.391, 38.379)	0.260 (0.009, 0.582)	0.005 (0.005, 0.110)	2.425 (1.513, 4.263)
	p value[2]		0.614	0.100	0.723	0.123	0.461	0.154
	SR		-0.076	-0.129	-0.027	-0.024	0.044	0.052
Plastic tableware	Not used	58	1.849 (0.856, 3.985)	0.587 (0.260, 1.992)	24.600 (8.771, 54.058)	0.331 (0.010, 0.741)	0.005 (0.005, 0.133)	2.525 (1.428, 3.813)
	Occasionally	68	1.48 (0.696, 3.670)	0.616 (0.055, 1.410)	14.388 (5.045, 33.396)	0.260 (0.006, 0.721)	0.005 (0.005, 0.080)	2.276 (1.483, 3.558)
	Daily used	26	2.332 (0.643, 3.947)	1 (0.010, 1.820)	14.272 (4.973, 28.375)	0.192 (0.010, 0.609)	0.005 (0.005, 0.100)	2.543 (1.658, 4.280)
	p value[2]		0.624	0.527	0.471	0.709	0.742	0.854
	SR		-0.049	-0.013	-0.143	-0.078	-0.044	0.005
Plastic wrap	Not used	94	1.480 (0.585, 3.893)	**0.553 (0.010, 1.233)**	14.797 (6.918, 40.267)	0.240 (0.010, 0.630)	0.005 (0.005, 0.103)	2.495 (1.470, 3.685)

	Occasionally	53	1.844 (0.842, 3.821)	**1.060 (0.165, 2.069)**	16.903 (4.548, 54.130)	0.390 (0.008, 0.768)	0.005 (0.005, 0.040)	2.280 (1.523, 3.932)
	Daily used	5	3.694 (1.557, 4.792)	**1.640 (0.636, 3.001)**	14.384 (8.205, 23.155)	0.490 (0.133, 1.790)	0.005 (0.005, 0.105)	2.410 (1.125, 3.620)
	p value[2]		0.559	0.155	0.984	0.422	0.633	0.958
	SR		0.073	**0.179**	0.033	0.106	-0.077	-0.028
Packaged Food for Microwave	Not used	39	1.744 (0.590, 6.592)	0.955 (0.010, 1.670)	18.000 (6.920, 51.260)	0.310 (0.010, 0.920)	0.005 (0.005, 0.140)	2.220 (1.307, 3.607)
	1-3 times/ month	64	1.757 (0.371, 3.852)	0.904 (0.272, 1.951)	17.313 (6.228, 48.567)	0.295 (0.010, 0.721)	0.005 (0.005, 0.110)	2.635 (1.674, 4.015)
	≥ 1 times/ week	49	1.650 (1.050, 3.524)	0.530 (0.090, 1.155)	14.286 (5.870, 25.260)	0.230 (0.010, 0.605)	0.005 (0.005, 0.080)	2.272 (1.465, 3.580)
	p value[2]		0.339	0.693	0.324	0.641	0.166	0.340
	SR		-0.016	-0.089	-0.128	-0.076	-0.106	0.029
Household items								
Air purifier	1-3 times/month	42	1.250 (0.510, 2.576)	0.681 (0.010, 1.780)	14.595 (7.905, 49.787)	0.425 (0.010, 1.332)	0.005 (0.005, 0.148)	2.475 (1.348, 3.535)
	1-3 times/week	38	2.262 (1.121, 4.633)	0.570 (0.160, 1.982)	24.979 (7.676, 44.561)	0.305 (0.010, 0.769)	0.005 (0.005, 0.085)	2.658 (1.490, 3.849)
	Daily	71	1.711 (0.660, 3.780)	0.813 (0.160, 1.670)	14.894 (4.100, 42.810)	0.259 (0.005, 0.617)	0.005 (0.005, 0.090)	2.220 (1.490, 3.440)
	p value[2]		0.083	0.909	0.668	0.394	0.579	0.881
	SR		0.065	0.000	-0.063	-0.140	-0.111	-0.012
Humidifier	1-3 times/ month	68	1.807 (0.615, 3.645)	0.631 (0.247, 1.702)	14.595 (7.083, 52.599)	**0.415 (0.010, 1.106)**	0.005 (0.005, 0.110)	2.360 (1.410, 3.659)

	1-3 times/ week	32	1.370 (0.790, 3.295)	0.844 (0.028, 1.408)	15.510 (5.820, 27.009)	**0.305 (0.010, 0.676)**	0.008 (0.005, 0.138)	2.855 (1.589, 3.915)
	Daily	50	1.986 (0.675, 4.000)	0.610 (0.010, 2.065)	17.782 (3.942, 42.93)	**0.169 (0.005, 0.497)**	0.005 (0.005, 0.055)	2.313 (1.517, 4.095)
	p value[2]		0.974	0.850	0.916	0.102	0.315	0.657
	SR		0.026	-0.007	-0.039	**-0.221**	-0.083	0.044
Air conditioner	1-3 times/ month	57	1.814 (0.773, 5.077)	0.480 (0.010, 2.034)	19.342 (10.005, 47.878)	0.260 (0.010, 0.723)	0.005 (0.005, 0.150)	2.910 (1.580, 4.385)
	1-3 times/ week	38	1.634 (0.668, 3.920)	0.725 (0.266, 1.570)	10.619 (3.248, 32.361)	0.305 (0.010, 0.703)	0.005 (0.005, 0.088)	2.011 (0.944, 3.198)
	Daily	55	1.611 (0.660, 2.581)	0.830 (0.100, 1.537)	14.894 (5.120, 45.230)	0.280 (0.005, 0.747)	0.005 (0.005, 0.080)	2.305 (1.530, 3.410)
	p value[2]		0.624	0.639	0.316	0.953	0.521	0.020
	SR		-0.101	0.024	-0.099	-0.017	-0.065	-0.115
Dehumidifier	1-3 times/ month	124	1.779 (0.800, 4.009)	0.635 (0.055, 1.710)	15.195 (6.685, 43.343)	0.267 (0.010, 0.724)	0.005 (0.005, 0.080)	2.527 (1.490, 3.905)
	1-3 times/ week	11	1.803 (0.437, 3.450)	0.810 (0.266, 2.370)	26.950 (5.360, 84.220)	0.440 (0.005, 0.891)	0.005 (0.005, 0.020)	2.262 (0.790, 2.800)
	Daily	16	1.410 (0.398, 2.443)	0.810 (0.208, 1.238)	17.892 (4.603, 38.231)	0.285 (0.147, 0.717)	0.020 (0.005, 0.115)	2.043 (1.565, 3.036)
	p value[2]		0.515	0.574	0.692	0.687	0.348	0.261
	SR		-0.116	0.045	0.023	0.042	0.043	-0.111
Mosquito repellent	Rarely used	122	1.596 (0.653, 3.462)	0.600 (0.065, 1.483)	14.930 (5.863, 42.948)	0.295 (0.010, 0.698)	0.005 (0.005, 0.100)	2.450 (1.486, 3.507)
	1-3 times/month	10	4.893 (3.038,	2.758 (1.198,	22.653 (17.117,	0.522 (0.196,	0.005 (0.005,	2.420 (1.994,

				7.861)		5.519)		41.279)		2.450)		0.108)		4.444)
	≥1 times/week	17	2.400	(0.910, 3.975)	0.830	(0.008, 1.500)	14.894	(5.050, 45.125)	0.143	(0.005, 1.380)	0.005	(0.005, 0.090)	2.650	(1.655, 4.140)
	p value[2]		0.152		0.026		0.267		0.388		0.966		0.528	
	SR		0.135		0.117		0.075		0.027		-0.014		0.073	
Odor neutralizer	Rarely used	95	1.711	(0.813, 3.760)	0.580	(0.050, 1.410)	14.700	(4.076, 43.360)	0.249	(0.010, 0.669)	0.005	(0.005, 0.110)	2.280	(1.488, 3.430)
	1-3 times/month	35	1.700	(0.787, 4.350)	1.060	(0.390, 2.922)	22.490	(8.234, 45.230)	0.517	(0.010, 0.891)	0.005	(0.005, 0.090)	2.650	(1.526, 4.510)
	≥1 times/week	18	2.115	(0.615, 2.880)	0.735	(0.054, 2.063)	12.280	(7.018, 24.317)	0.345	(0.058, 0.663)	0.005	(0.005, 0.055)	2.864	(1.600, 4.829)
	p value[2]		0.947		0.163		0.293		0.440		0.615		0.621	
	SR		-0.012		0.134		0.056		0.102		-0.076		0.115	
Bathroom Disinfectant	Rarely used	37	1.550	(0.462, 3.215)	0.630	(0.170, 1.604)	18.060	(8.110, 49.110)	0.180	(0.005, 0.490)	0.005	(0.005, 0.080)	2.140	(1.332, 3.793)
	1-3 times/month	76	1.757	(0.915, 4.228)	0.825	(0.124, 1.766)	15.545	(3.680, 42.390)	0.295	(0.010, 0.734)	0.005	(0.005, 0.110)	2.527	(1.618, 3.973)
	≥1 times/week	38	2.310	(0.758, 3.808)	0.572	(0.010, 1.634)	15.421	(7.238, 42.509)	0.435	(0.009, 0.950)	0.005	(0.005, 0.093)	2.106	(1.324, 3.217)
	p value[2]		0.852		0.853		0.406		0.177		0.512		0.276	
	SR		0.066		-0.007		-0.042		0.145		-0.064		-0.043	
Dietary Habits (consumption)														
Canned Tuna	1-3 times/month	126	1.706	(0.777, 3.790)	0.681	(0.145, 1.494)	14.802	(6.023, 41.576)	0.305	(0.010, 0.760)	0.005	(0.005, 0.103)	2.445	(1.513, 3.798)
	≥1 times/week	20	1.880	(0.315,	0.780	(0.104,	17.207	(7.763,	0.080	(0.010,	0.005	(0.005,	2.477	(1.520,

				4.198)		2.225)		43.363)		0.510)		0.085)		3.900)
	p value[1]		0.671		0.890		0.561		0.205		0.584		0.774	
	SR		0.002		0.0321		0.041		-0.104		-0.039		0.017	
Canned Food	0	38	2.097	(1.213, 6.247)	0.714	(0.175, 1.555)	12.798	(5.103, 33.47)	0.300	(0.009, 0.835)	0.005	(0.005, 0.093)	2.256	(1.428, 3.452)
	1-3 times/month	77	1.300	(0.570, 3.366)	0.610	(0.010, 1.655)	17.723	(7.678, 50.028)	0.280	(0.010, 0.719)	0.005	(0.005, 0.090)	2.440	(1.485, 3.815)
	≥1 times/week	37	1.844	(1.102, 3.943)	0.813	(0.320, 2.024)	14.286	(3.385, 26.537)	0.273	(0.010, 0.667)	0.005	(0.005, 0.110)	2.824	(1.520, 3.801)
	p value[2]		0.444		0.255		0.356		0.790		0.916		0.628	
	SR		-0.047		0.025		-0.021		-0.028		-0.020		0.082	
Hamburger	0	23	1.844	(0.570, 8.140)	0.621	(0.010, 1.960)	16.672	(7.200, 41.165)	**0.080**	**(0.005, 0.556)**	0.020	(0.005, 0.140)	3.080	(1.440, 4.07)
	1-3 times/month	111	1.650	(0.787, 3.650)	0.640	(0.100, 1.640)	15.160	(6.000, 46.540)	**0.380**	**(0.010, 0.760)**	0.005	(0.005, 0.080)	2.380	(1.526, 3.607)
	≥1 times/week	18	1.957	(0.622, 4.723)	0.685	(0.149, 1.910)	14.542	(5.825, 27.890)	**0.074**	**(0.005, 0.497)**	0.005	(0.005, 0.100)	2.750	(1.401, 3.808)
	p value[2]		0.928		0.991		0.842		**0.017**		0.282		0.919	
	SR		-0.022		-0.030		-0.058		0.011		-0.099		-0.025	
French Fries	0	40	2.242	(0.470, 3.612)	0.580	(0.010, 1.719)	13.425	(5.355, 39.578)	0.275	(0.010, 0.704)	0.005	(0.005, 0.093)	2.407	(1.489, 3.988)
	1-3 times/month	101	1.580	(0.807, 3.830)	0.810	(0.130, 1.655)	17.142	(7.246, 44.295)	0.320	(0.010, 0.743)	0.005	(0.005, 0.090)	2.429	(1.465, 3.529)
	≥1 times/week	11	1.883	(0.140, 4.362)	0.813	(0.380, 1.863)	14.700	(1.264, 31.870)	0.280	(0.005, 0.858)	0.005	(0.005, 0.130)	2.220	(1.490, 4.966)

	p value[2]		0.770		0.801		0.543		0.899		0.816		0.994	
	SR		-0.036		0.031		0.038		-0.003		-0.009		-0.013	
Pizza	0	23	1.814	(1.239, 5.330)	0.560	(0.180, 1.420)	9.840	(2.520, 34.818)	0.200	(0.005, 0.594)	0.005	(0.005, 0.140)	2.272	(1.488, 3.910)
	1-3 times/month	122	1.728	(0.729, 3.765)	0.825	(0.093, 1.965)	16.159	(7.515, 45.558)	0.315	(0.010, 0.760)	0.005	(0.005, 0.080)	2.420	(1.473, 3.685)
	≥1 times/week	6	1.040	(0.190, 6.292)	0.370	(0.009, 5.340)	20.275	(11.341, 27.441)	0.145	(0.009, 0.347)	0.008	(0.005, 0.100)	3.425	(2.110, 5.152)
	p value[2]		0.517		0.871		0.915		0.216		0.354		0.346	
	SR		-0.081		0.018		0.057		0.041		-0.078		0.045	
Chicken	0	16	1.480	(0.527, 6.186)	0.360	(0.006, 1.238)	12.975	(3.937, 69.798)	0.245	(0.010, 0.954)	0.050	(0.005, 0.193)	2.750	(1.528, 3.785)
	1-3 times/month	122	1.596	(0.653, 3.535)	0.681	(0.145, 1.887)	15.195	(6.843, 45.558)	0.310	(0.010, 0.741)	0.005	(0.005, 0.055)	2.350	(1.488, 3.728)
	≥1 times/week	12	2.981	(1.318, 6.391)	0.572	(0.098, 1.943)	20.795	(15.456, 29.77)	0.270	(0.010, 0.510)	0.050	(0.005, 0.123)	2.854	(1.563, 4.523)
	p value[2]		0.286		0.415		0.969		0.863		0.090		0.895	
	SR		0.058		0.073		0.040		-0.021		-0.031		0.002	
Food Delivery	0	10	3.425	(0.493, 11.033)	1.294	(0.843, 3.747)	19.425	(0.838, 36.405)	0.120	(0.005, 1.136)	**0.005**	**(0.005, 1.075)**	2.472	(1.120, 4.541)
	1-3 times/month	62	1.525	(0.559, 2.885)	0.626	(0.123, 1.413)	14.235	(5.103, 41.670)	0.267	(0.010, 0.762)	**0.005**	**(0.005, 0.115)**	2.278	(1.382, 4.028)
	≥1 times/week	80	1.843	(0.803, 3.882)	0.582	(0.055, 1.936)	17.782	(7.407, 47.431)	0.331	(0.010, 0.695)	**0.005**	**(0.005, 0.038)**	2.470	(1.652, 3.568)
	p value[2]		0.420		0.106		0.236		0.677		**0.049**		0.514	

	SR		0.017	-0.063	0.111	0.038	**-0.164**	0.066
Popcorn and Nacho	0	132	1.631 (0.665, 3.850)	0.815 (0.163, 1.710)	15.027 (6.913, 43.343)	0.267 (0.010, 0.695)	**0.005 (0.005, 0.078)**	2.470 (1.482, 3.833)
	1-3 times/month	17	1.814 (0.865, 3.695)	0.590 (0.142, 0.981)	16.903 (3.572, 45.105)	0.410 (0.020, 2.152)	**0.050 (0.005, 0.172)**	2.030 (1.470, 2.925)
	≥1 times/week	3	3.780 (1.020, -)	0.010 (0.005, -)	19.100 (9.460, -)	0.290 (0.260, -)	**0.090 (0.010, -)**	1.930 (1.780, -)
	p value[2]		0.762	0.415	0.953	0.426	0.185	0.803
	SR		0.046	-0.055	-0.017	0.105	**0.172**	-0.072
Electronic devices								
Mobile Phone	4-6 times/week	21	1.940 (0.190, 3.770)	1.250 (0.390, 2.837)	16.573 (1.707, 68.254)	0.351 (0.005, 0.803)	0.005 (0.005, 0.100)	2.190 (1.297, 2.527)
	<1 hour/day	71	1.567 (0.800, 4.010)	0.630 (0.100, 1.480)	14.490 (7.200, 41.130)	0.320 (0.010, 0.707)	0.005 (0.005, 0.080)	2.698 (1.488, 3.710)
	≥1 hour/day	60	1.864 (0.713, 3.683)	0.587 (0.020, 1.342)	16.565 (5.188, 43.170)	0.204 (0.010, 0.627)	0.005 (0.005, 0.115)	2.343 (1.498, 3.905)
	p value[2]		0.513	0.308	0.696	0.835	0.836	0.878
	SR		0.035	-0.109	0.008	-0.057	0.088	0.044
TV	4-6 times/week	25	1.957 (0.829, 8.414)	0.690 (0.228, 1.947)	27.030 (7.606, 49.787)	0.275 (0.005, 0.516)	0.013 (0.005, 0.095)	1.992 (1.185, 3.400)
	<1 hour/day	31	1.500 (0.800, 3.450)	0.880 (0.160, 1.980)	14.490 (6.910, 53.867)	0.351 (0.010, 0.760)	0.005 (0.005, 0.080)	2.440 (1.400, 3.780)
	≥1 hour/day	95	1.844 (0.680, 3.694)	0.583 (0.050, 1.537)	15.160 (4.100, 37.450)	0.260 (0.010, 0.739)	0.005 (0.005, 0.100)	2.514 (1.490, 3.850)

	p value[2]		0.754	0.592	0.306	0.974	0.443	0.340
	SR		-0.017	-0.069	-0.086	0.033	-0.060	0.099
PC	4-6 times/week	64	1.675 (0.696, 3.743)	0.676 (0.025, 1.840)	15.844 (5.188, 45.608)	**0.193 (0.005, 0.619)**	0.005 (0.005, 0.088)	2.313 (1.450, 3.433)
	<1 hour/day	16	0.699 (0.300, 3.696)	0.563 (0.016, 2.714)	14.133 (7.652, 54.381)	**0.010 (0.005, 0.657)**	0.005 (0.005, 0.148)	1.900 (1.327, 3.902)
	≥1 hour/day	70	2.192 (1.016, 3.884)	0.817 (0.269, 1.502)	17.198 (7.130, 40.259)	**0.385 (0.142, 0.874)**	0.005 (0.005, 0.085)	2.497 (1.535, 3.860)
	p value[2]		0.152	0.901	0.970	**0.015**	0.838	0.835
	SR		0.059	0.004	0.016	**0.198**	0.029	0.053
Tablet PC	<1 hour/day	13	2.690 (1.305, 5.217)	1.060 (0.725, 4.409)	15.230 (1.076, 27.95)	0.203 (0.005, 1.050)	0.005 (0.005, 0.120)	**1.330 (0.748, 2.847)**
	≥1 hour/day	12	1.425 (0.443, 1.797)	0.641 (0.044, 1.235)	17.259 (9.078, 92.168)	0.270 (0.006, 1.927)	0.005 (0.005, 0.018)	**2.068 (1.424, 3.902)**
	≥3 hour/day	127	1.803 (0.630, 3.780)	0.580 (0.050, 1.780)	14.894 (6.910, 43.360)	0.290 (0.010, 0.694)	0.005 (0.005, 0.100)	**2.509 (1.530, 3.850)**
	p value[2]		0.170	0.060	0.122	0.753	0.564	**0.021**
	SR		-0.044	-0.097	0.014	0.017	0.060	**0.177**

The bold font indicates statistical significance. EDCs levels are presented as median and interquartile range. [1] *p* value was calculated using the Mann-Whitney test. [2] *p* value was calculated using the Kruskal-Wallis test. EDCs, endocrine disrupting chemicals; BPA, bisphenol-A; MEP, monoethyl phthalate; PFOA, perfluorooctanoic acid; SR, Spearman's rho.

Table S3. Linear associations between EDCs and birthweight according to the presence or absence of GDM using univariable linear regression analysis

		**Total**	**GDM**	**Non-GDM**
**Birthweight percentile**		**β (95% CI)**	**β (95% CI)**	**β (95% CI)**
BPA	2nd trimester urine	-0.480 (-2.446, 1.487)	1.418 (-2.146, 4.983)	-1.083 (-3.429, 1.263)
	3rd trimester urine	0.990 (-0.725, 2.705)	3.844 (1.043, 6.645)	-0.0241 (-2.386, 1.905)
	Cord blood	-0.609 (-1.943, 0.725)	0.815 (-1.501, 3.131)	-1.128 (-2.807, 0.551)
MEP	2nd trimester urine	0.968 (-1.399, 3.336)	2.395 (-1.639, 6.428)	0.295 (-2.615, 3.206)
	3rd trimester urine	0.503 (-0.951, 1.957)	0.278 (-1.881, 2.437)	0.794 (-1.199, 2.787)
	Cord blood	0.345 (-1.068, 1.757)	1.650 (-0.772, 4.073)	-0.140 (-1.902, 1.622)
PFOA	3rd trimester urine	-0.570 (-2.296, 1.156)	-0.019 (-2.944, 2.906)	-1.042 (-3.217, 1.133)
	Cord blood	2.066 (-2.975, 7.107)	-5.183 (-13.936, 3.570)	5.354 (-0.791, 11.499)

EDC, endocrine disrupting chemiclas; GDM, gestational diabetes mellitus; BPA, bisphenol-A; MEP, monoethyl phthalate; PFOA, perfluorooctanoic acid

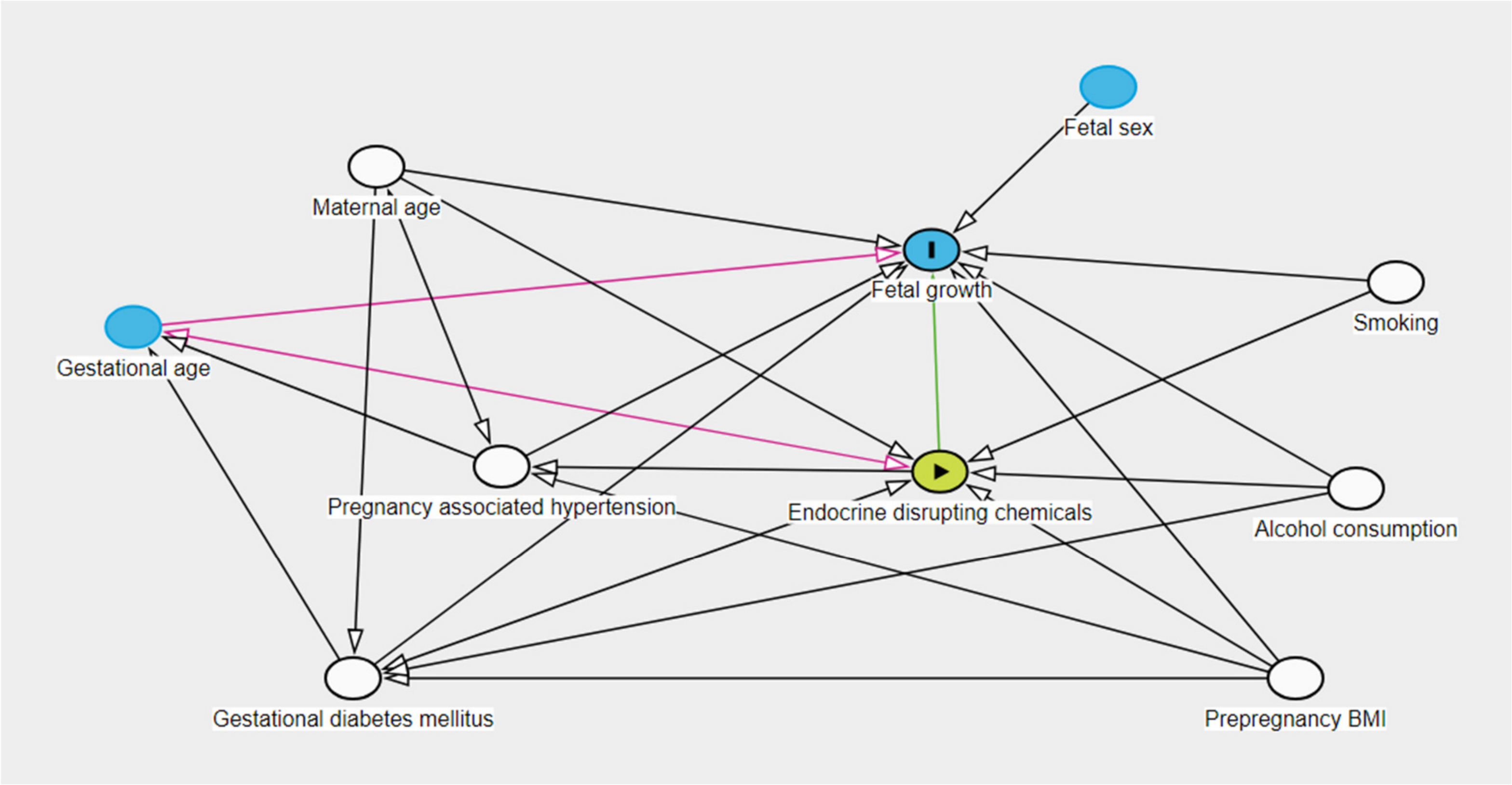

Figure S1 Directed acyclic graphs showing the relationship of prenatal EDC exposures and fetal growth, and covariates.

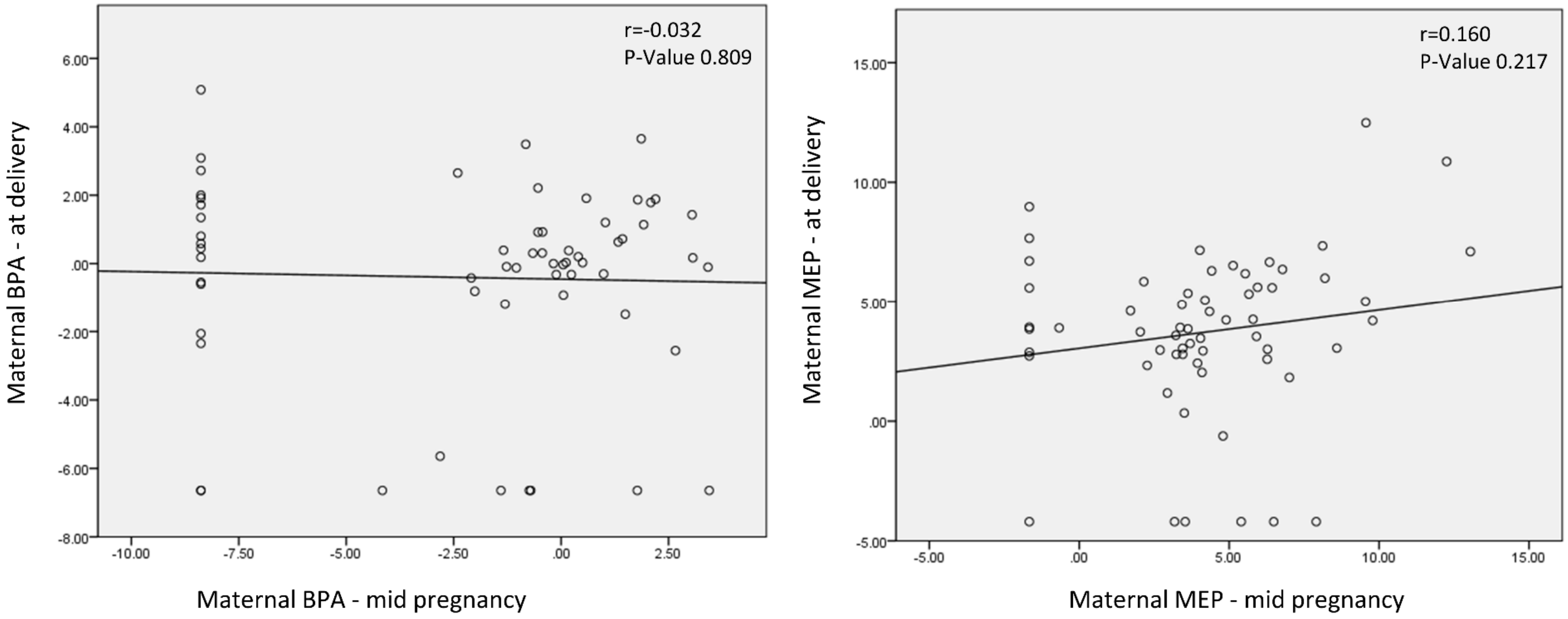

Figure S2 Pearson correlations between Log transformed values of BPA and MEP in maternal urine at second trimester and before delivery. Both BPA and MEP showed no correlation between their levels during second trimester and before delivery.
